# A new estimation of the total flavonoids in silkworm cocoon sericin layer through aglycone determination by hydrolysis-assisted extraction and HPLC-DAD analysis

**DOI:** 10.3402/fnr.v60.30932

**Published:** 2016-03-14

**Authors:** Jin-Ge Zhao, Yu-Qing Zhang

**Affiliations:** Silk Biotechnology Laboratory, School of Biology and Basic Medical Sciences, Soochow University, Suzhou 215123, China

**Keywords:** silkworm cocoon, total flavonoids, aglycones, quercetin, kaempferol, hydrolysis, extraction

## Abstract

**Background:**

Silk sericin and a few non-protein components isolated from the cocoon layer including two silk proteins in silkworm *Bombyx mori* has many bioactivities. The dietary sericin possess antinatural oxidation, anticancer, antihyperlipidemic, and antidiabetic activities. The non-protein components surrounding the sericin layer involve in wax, pigments mainly meaning flavonoids, sugars, and other impurities. However, very few investigations have reported the estimation of the total flavonoids derived from the cocoon layer. The flavonoids are commonly present in their glycosylated forms and mostly exist as quercetin glycosides in the sericin layers of silkworm cocoons.

**Objective:**

The aim of this study was to find a more accurate method to estimate the level of the total flavonoids in silkworm cocoons.

**Design:**

An efficient procedure of hydrolysis-assisted extraction (HAE) was first established to estimate the level of the total flavonoids through the determination of their aglycones, quercetin, and kaempferol. Then, a comparison was made between traditional colorimetric method and our method. In addition, the antioxidant activities of hydrolysis-assisted extract sample were determined.

**Results:**

The average contents of quercetin and kaempferol were 1.98 and 0.42 mg/g in Daizo cocoon. Their recoveries were 99.56 and 99.17%. The total sum of quercetin and kaempferol was detected to be 2.40±0.07 mg/g by HAE-HPLC, while the total flavonoids (2.59±0.48 mg/g) estimated by the traditional colorimetric method were only equivalent to 1.28±0.04 mg/g of quercetin. The HAE sample also exhibits that IC_50_ values of scavenging ability of diphenyl picryl hydrazinyl (DPPH) radical and hydroxyl radical (HO·) are 243.63 µg/mL and 4.89 mg/mL, respectively.

**Conclusions:**

These results show that the HAE-HPLC method is specificity of cocoon and far superior to the colorimetric method. Therefore, this study has profound significance for the comprehensive utilization of silkworm cocoon and also may be applied to the estimation of total flavonoids in other functional foods.

Silk, derived from the silkworm *Bombyx mori*, is a natural protein that is mainly made of sericin and fibroin proteins. Sericin envelops the fibroin fiber with successive sticky layers that help in the formation of a cocoon. Sericin ensures the cohesion of the cocoon by gluing silk threads together (1). The cocoon shell of the silkworm is composed of 70% silk fibroin fibers, 25% sericin, and 5% non-sericin components, with the sericin and non-sericin components concentrated in a layer surrounding the silk fibroin (2). Sericin has many bioactivities, including antioxidant (3), skin care (4), UV protection (5), and antitumor (6) activities and cell mitosis promotion (7); it is also used as a cell culture matrix (8). It has been reported that sericin as a food additive has many biological properties. Siqin et al. found that dietary sericin suppresses development of colon tumors by reducing oxidative stress, cell proliferation, and nitric oxide production (9). Some researchers also reported the usefulness of sericin for improving the lipid and carbohydrate metabolism in rats fed on a high-fat diet (10), and a dietary sericin protects mice against diabetic complication (11).

The non-sericin components primarily consist of an ethanol extract from the sericin layer of the silkworm cocoon, which was found to mainly contain flavonoids and free amino acids. Tamura et al. identified three flavonoid 5-glucosides in the sericin layer of *B. mori* cocoons; these were quercetin 5,4′-di-O-β-d-glucopyranoside, quercetin 5,7,4′-tri-O-β-d-glucopyranoside, and the known quercetin 5-O-β-d-glucopyranoside (12). Kurioka et al. also purified and identified seven flavonoids from the yellow-green cocoon shell of the Sasammayu silkworm (a hybrid of Daizo) (13). Three quercetin glycosides (quercetin 5-*O-*β-d-glucoside, quercetin 7-*O-*β-d-glucoside, and quercetin 4′-*O-*β-d-glucoside), two kaempferol glycosides (kaempferol 5-*O-*β-d-glucoside and kaempferol 7-*O-*β-d-glucoside), and their aglycones, quercetin, and kaempferol were isolated from an ethanolic extract of the cocoon shells. In addition, the l-proline moieties prolinalin A (6-C-(2*S*, 5*S*)-prolin-5-yl quercetin) and prolinalin B (6-C-(2*S*, 5*R*)-prolin-5-yl quercetin) were isolated from the *B. mori* cocoon shell (14). These flavonoids also possess anticancer, hypolipidemic, antiaging, and anti-inflammatory activities. Very few experiments have been carried out using high purity sericin samples, especially those purified by the ethanol precipitation method. It is unclear whether the above biological activities can be attributed solely to the sericin protein or to the joint effect of sericin and the non-sericin components. Therefore, it is imperative to find a more accurate method to estimate the level of the total flavonoids in silkworm cocoons.

The amount of the total flavonoids was estimated conventionally by a colorimetric method using rutin as a standard. Recently, the colorimetric method has come under criticism for having large errors and boundedness in estimating the amount of total flavonoids in biosamples (15). Therefore, a hydrolysis-assisted extraction (HAE) was constructed in this paper to release aglycones from the flavonoid glycosides in the biosamples. Two flavonoid aglycones, quercetin and kaempferol, only present in the Daizo cocoon shell, can be detected quantitatively by high performance liquid chromatography with a diode array detector (HPLC-DAD). Then, the total amount of the two aglycones is used to estimate or express the total flavonoids and their bioactivities in biosamples especially in silkworm cocoons.

## Materials and methods

### Chemicals and materials

Daizo cocoons, commercially common white cocoons and other colored cocoons (Supplementary Fig. 1) from strains of the silkworm *B. mori* were provided by the Sericulture Institute at Soochow University. The rutin, quercetin, and kaempferol standards were purchased from Shanghai Chemical Reagent Co. Ltd (Shanghai, China). All of the other chemicals and solvents used were of analytical grade, except those used for the HPLC analysis, such as acetonitrile, which were HPLC grade.

### HAE of silkworm cocoons

The Daizo cocoon shell was cut into pieces, and 250-mg pieces were suspended in 10 ml of an organic solvent–acid–water (v/v/v) solution and kept at 75°C for 2 h for the hydrolysis. The organic solvents tested include methanol, ethanol, or acetone; the acid used was HCl or H_2_SO_4_; and the water was acidic electrolyzed water (pH2.5) or alkaline electrolyzed water (pH11.5). To ensure the volume of the hydrolysis solution after the experiment was the same as before the experiment, the volume of the active solution was quantified by addition of the same mixed solution of organic solvent–acid–water. Then, the supernatant after hydrolysis was used in the following analysis.

### Standard curves

The standard samples, quercetin and kaempferol, were dissolved quantitatively in methanol. Two different standard sample solutions were combined to make a mixed solution with five concentrations for HPLC analysis. Ten microliters of each concentration was injected into the liquid chromatographer and each sample was detected three times.

### Reproducibility analysis

A 250 mg sample of cocoon pieces was suspended in 10 ml of a methanol–HCL–water (7:2:1, v/v/v) solution and kept at 75°C for 4 h. After treatment, the volume of the reactive solution was quantified to be the same as before the treatment. Five repeated extractions of the samples were measured by HPLC-DAD to calculate the relative standard deviation (RSD).

### Recovery assay

Known amounts of quercetin and kaempferol were added into the cocoon sample solution; then the mixture was hydrolyzed and extracted. The extraction supernatant was detected quantitatively by HPLC-DAD. The recoveries of the known amount of the two aglycones were calculated by the linear equation produced from the standard curves.

### Assay of total flavonoids by colorimetric method

A colorimetric method with rutin as a standard was used conventionally for the determination of total flavonoid content (16, 17). Ten milligrams of the sample was accurately taken, 1.0 mg/mL of an aqueous solution was prepared, and 1.0 mL of the solution was accurately taken and added into the test tube. The samples were replicated three times. According to the steps of the standard curve, the absorbance at a wavelength of 510 nm was measured, and the content of the total flavonoids was calculated. In addition, the value of the total flavonoids was converted into an equivalent quantity of quercetin, according to the molecular mass of rutin. In this case, it is beneficial to compare the studies and analysis between the two methods.

### HPLC-DAD analysis

A previously reported method was slightly modified for HPLC analysis (18). The Shimazu HPLC system consisted of a pump (LC-20AT), a diode array detector (DAD, SPD-M20A), and a 300SB-C18 column (Agilent 250×4.6 mm). The sample solution was filtered through a 0.45-µm nylon membrane filter (Millipore), and 10 µL was injected into the liquid chromatographer. The mobile phase was methyl alcohol–water–phosphoric acid, with a ratio of 500:500:0.4 (v/v/v). The flow rate was 1 ml/min, and the eluate absorbance was monitored at 370 nm using a scanning range of 200–600 nm.

### Evaluation of antioxidant activity in vitro

The hydrolysis liquid from 200 g Daizo cocoons was adjusted to pH3.5 using NaOH. Then, the concentrated solvents got through D101 macroporous resin (Shanghai, China). The D101 resin was washed with distilled water in order to remove NaCl, and then the purified sample subsequently recovered with gradient elution. The elute was concentrated in vacuo, freeze-dried, and referred to as the hydrolysis-assisted extract sample (HAEs).

Diphenyl picryl hydrazinyl (DPPH) radical scavenging activities of HAEs were measured using the method of Zhao (19).

The scavenging activity of HAEs on hydroxyl radical (HO·) was determined according to the previous method (20).

### Statistical analysis

The data were expressed as the mean±standard deviation (SD). Comparisons were made using one-way ANOVA with the Origin8.5 software. A value of *p*<0.05 was considered statistically significant.

## Results

### Identification and determination of quercetin and kaempferol

**Fig. 1:**
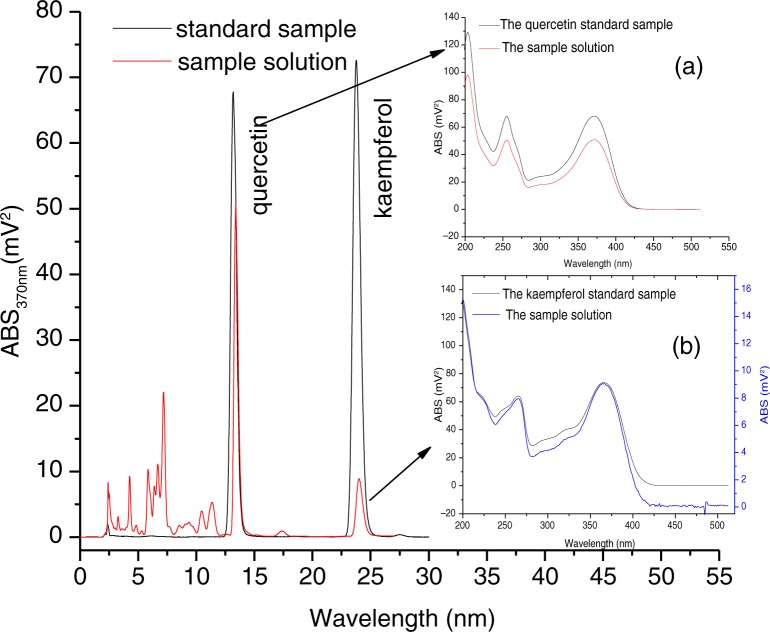
HPLC chromatogram of the standard sample and the extract of a cocoon. (a) The UV-VIS absorption spectra corresponding to the quercetin peak (quercetin standard sample and the extract of cocoon). (b) The UV-VIS absorption spectra corresponding to the kaempferol peak (kaempferol standard sample and the extract of cocoon).

The concentrations and the area under the chromatographic peak showed a good linear relationship. On a C18 reverse-phase column, quercetin and kaempferol showed good retention behavior and two peaks at retention times of 13.17 and 23.75 min by the HPLC-DAD analysis. The HPLC chromatographic behavior of the methanol extract is shown in Fig. 1. The retention times of these peaks at 13.39 and 24 min were very close to the retention times of the standard samples. The results also show that the peak UV absorption spectrum of the methanol extract was almost the same as that of the standard sample with a maximum absorption peak at 370 nm. This demonstrates that the two peaks of the extract were the two aglycones: quercetin and kaempferol.

The linear regression equations for the two standard samples were as follows: quercetin, *y=*110311+2599690*x, R=*0.99289; and kaempferol, *y=*15655.5+3452790*x*, *R=*0.99464. The amounts of the two aglycones detected by HPLC-DAD were calculated individually by the two linear regression equations in the following experiment.

### The optimum of HAE condition

**Fig. 2:**
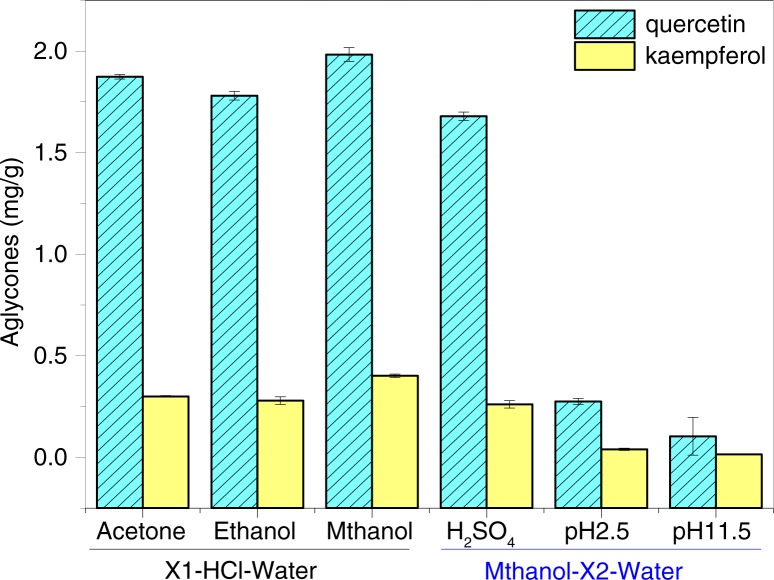
Effects of the extraction solvents in an organic solvent–acid–H_2_O mixture solution (7:1:2, v/v/v) on the extraction efficiency of the two aglycones from Dazio cocoons. X1 means acetone, ethanol, or methanol; X2 means H_2_SO_4_, acidic electrolyzed water (pH2.5), or alkaline electrolyzed water (pH11.5).

The selection of solvents can play an important role in the extraction of phenolic and antioxidant compounds from complex samples, but the most widely used solvents for extracting phenolic substances are methanol and methanol/water mixtures (21). In our study, methanol, ethanol, and acetone were used for the optimization experiment of organic solvent–acid–water in HAE. The left side of Fig. 2 shows that methanol was better than the two others in the extraction of quercetin and kaempferol from Daizo cocoons under the same mixture ratio (organic solvent–HCl–water, 7:1:2, v/v/v) and extraction conditions (hydrolysis at 75°C for 2 h). The two aglycones, quercetin and kaempferol, from Daizo cocoons could be detected to be near 2.0 mg/g and approximately 0.45 mg/g, respectively. Therefore, methanol was used in the next HAE optimization test. Our group also reported that the acidified electrolyzed water was used to extract anthocyanin from purple sweet potato (22). Here, the acidic or alkaline electrolyzed water has been used for the HAE test. The results showed that the extraction efficiency of the two aglycones in acidic electrolyzed water (pH2.5) or strongly alkaline electrolyzed water (pH11.5) was very low using the same cocoons under the same extraction conditions (the right side of Fig. 2). In addition, when H_2_SO_4_ was added into the extraction system (methanol–H_2_SO_4_–water, 7:1:2, v/v/v), a better extraction efficiency of the two aglycones from the cocoon was observed.

**Fig. 3:**
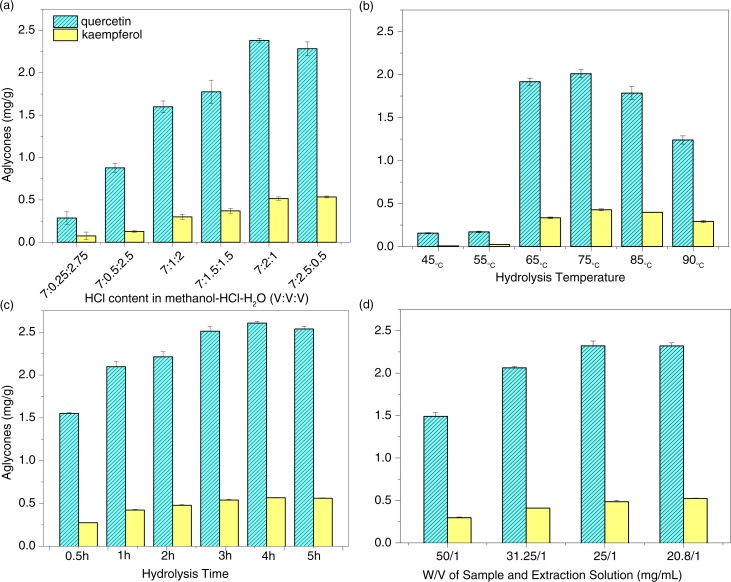
Effects of HCl concentration in (a) methanol–HCl–H_2_O, (b) hydrolysis temperature and (c) time, and (d) w/v ratio of sample/extraction solution on the extraction efficiency of two aglycones, quercetin and kaempferol, from Daizo cocoons.

To optimize the extraction conditions, we investigated the effects of HCl concentration in methanol–HCl–H_2_O, hydrolysis temperature and time, and the w/v ratio of the sample to extraction solution on the extraction efficiency of two aglycones, quercetin and kaempferol, from Daizo cocoons. First, the effects of the solvent ratio, especially HCl concentration, in the ternary system of methanol–HCl–H_2_O on the hydrolysis and extraction of the two flavonols from cocoons were studied (Fig. 3a). As shown in the figure, the extraction efficiency of the two aglycones rose with the increase of HCl concentration. When the mixture ratio of HCl concentration rose to 7:2.5:0.5, the extraction efficiency of the two aglycones did not rise but decreased slightly, indicating that the hydrolysis and extraction abilities for the two aglycones were the highest at the 7:2:1 ratio. It implied that a higher HCl concentration than the 7:2:1 mixture ratio may destroy the structure or characteristics of the flavonoid aglycones. Therefore, the mixture ratio of the ternary system of methanol–HCl–H_2_O (7:2:1, v/v/v) is always used for the following hydrolysis and extraction experiments.

Second, we also investigated effects of the hydrolysis temperature and time in the methanol–HCl–H_2_O (7:2:2, v/v/v) on the extraction efficiency of the two aglycones from Daizo cocoons. As shown in Fig. 3b, the extraction amounts of these aglycones were very low at 45°C and 55°C. When the extraction temperature reached 65°C, their extraction efficiencies rose rapidly. When the temperature was 75°C, the highest extraction rate could be observed. When the temperature rose again, the extraction efficiency did not increase but decreased. This situation seems to be the same as the elevated HCl concentration. Figure 3c showed effects of the extraction temperature on the extraction efficiency of the two aglycones from cocoons. The amount of the flavonol aglycones gradually rose with the increase in extraction time; it achieved an optimal value at 75°C and 4 h, and subsequently, it began to decrease slightly.

Third, the effect of the solid-to-solvent ratio on the extraction efficiency of the two flavonol aglycones from cocoons was also studied with four ratios (50:1, 31.25:1, 25:1, 20.8:1; w/v) over a 4-h extraction period with a 7:2:1 mixture solution (methanol–HCL– water, v/v/v) at 75°C. A marked increase in the responses of the extract was observed up to 25:1 solid-to-solvent ratio (Fig. 3d). To minimize the solvent requirement without compromising the responses, a solid-to-solvent ratio of 25:1 (mg:mL) was selected in the following extraction experiments except where otherwise noted.

### Reproducibility for aglycone extraction and determination by HAE-HPLC

The sample solution was extracted five times, and then, these samples were measured by HPLC-DAD (Table 1). According to their individual linear equation, the contents of quercetin and kaempferol were calculated to be an average of 1.98 and 0.42 mg/g with an RSD of 3.01 and 2.38%, respectively. These data indicate that the extraction and determination of the HAE and HPLC-DAD for the quercetin and kaempferol from cocoons shows good reproducibility.

**Table 1:** Reproducibility for the determination of the two aglycones by HAE-HPLC method

Aglycones	Repeated measurements (mg/g)					Means (mg/g)	±SD	RSD (%)
Quercetin	1.96	1.96	2.00	1.96	2.07	1.98	0.06	3.01
Kaempferol	0.42	0.42	0.42	0.41	0.44	0.42	0.01	2.38

Experimental materials: Daizo cocoons.

### Recovery of aglycone extraction by HAE-HPLC method

To determine the extraction recoveries of the two aglycones, a known content of quercetin and kaempferol were added to the mixed solution of sample and extraction solvent prior to hydrolysis and extraction. The contents of quercetin and kaempferol were calculated by the above individual linear equation produced from the standard samples. The experimental results showed that the extraction recovery rate for quercetin and kaempferol were 99.56%, with an RSD of ±1.69%, and 99.17%, with an RSD of ±1.57% (Table 2). This is a high recovery, bearing in mind the complexity of the analyses. The recovery test showed that the HAE method for the hydrolysis and extraction of quercetin and kaempferol from Daizo cocoons exhibits very good precision.

**Table 2:** Recoveries for the extraction of the two aglycones by HAE-HPLC method

Aglycones	Sample content (mg/g)	Dosage (mg)	Theoretical value (mg/g)	Measured value (mg/g)	Recovery (%)	RSD (%)
Quercetin	1.38±0.02	1	2.37	2.38±0.04	99.56	1.69
Kaempferol	0.24±0.01	0.2	0.44	0.44±0.01	99.17	1.57

Experimental materials: Daizo cocoons.

### A comparison with the traditional colorimetric method

To verify whether the HAE-HPLC method is comparable with the traditional UV colorimetric method, we used various silkworm cocoons, including colored or white cocoons, to analyze the contents of the two aglycones and compare them with each other (Table 3). There was a great difference in the amount of total flavonoids using various silkworm cocoons. It was found that colored cocoon 1-427 has the highest sum (3.22 mg/g) of the two aglycones of all silkworm cocoons using the HAE-HPLC method, but only 1.02 mg/g of quercetin in the same cocoon has been detected by the traditional colorimetric method. The two aglycones had almost not been detected in white cocoons by using the former method, while 0.24 mg/g of quercetin has been detected by the later method. A Daizo cocoon shell had a 2.4 mg/g sum of quercetin (1.98 mg/g) and kaempferol (0.42 mg/g) using the former method, while it had 1.28 mg/g of quercetin using the colorimetric method, only half of the detected value above. These results show that the values measured using the traditional colorimetric method is not able to reflect the real amount of total flavonoids in silkworm cocoons.

**Table 3:** The content of total flavonoids in silkworm cocoons by the two methods (mg/g ±SD)

		Daizo	White	Colored cocoons								
				
Methods	Aglycones	Cocoon	Cocoon	1201	1-342	1-425	1-427	1-451	1-474	1-901	1-949	4-524
HAE-HPLC	Quercetin	1.98	0.02	0.04	1.41	0.81	2.78	0.04	0.04	0.22	2.38	1.57
	(Q)	±0.06	±0.01	±0.01	±0.08	±0.02	±0.33	±0.03	±0.01	±0.01	±0.12	±0.13
	Kaempferol	0.42	0	0.02	0.22	0.18	0.44	0.02	0.03	0.06	0.37	0.33
	(K)	±0.01	0	±0.01	±0.01	±0.02	±0.04	±0.01	±0.01	±0.01	±0.02	±0.01
	Total sum	2.40	0.02	0.05	1.63	0.99	3.22	0.06	0.07	0.28	2.75	1.89
	(Q + K)	±0.07	±0.01	±0.01	±0.08	±0.03	±0.31	±0.01	±0.01	±0.01	±0.11	±0.12
Colorimetry	Total flavonoids	2.59	0.48	0.43	2.79	3.23	2.06	1.01	0.69	2.07	2.90	1.07
	(Rutin)	±0.09	±0.12	±0.25	±0.19	±0.37	±0.22	±0.18	±0.10	±0.20	±0.01	±0.1
	≈	1.28	0.24	0.21	1.38	1.60	1.02	0.50	0.34	1.02	1.44	0.53
	Quercetin	±0.04	±0.06	±0.12	±0.09	±0.18	±0.11	±0.09	±0.05	±0.10	±0.01	±0.05

Q + K is quercetin + kaempferol, ‘≈Quercetin’ means that the value of total flavonoids by the UV colorimetric method was converted into an equivalent quantity of quercetin according to the molecular mass of rutin.

**Fig. 4:**
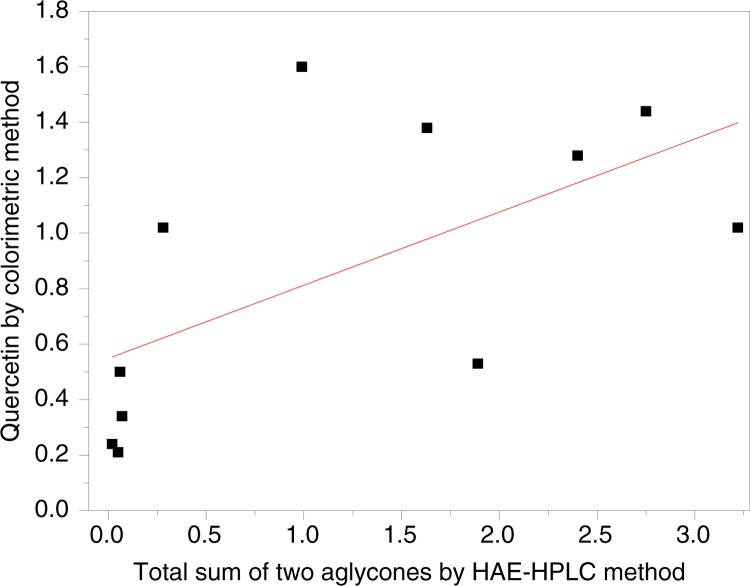
The correlation between the total amount of quercetin and kaempferol detected by HAE-HPLC and the equivalent quantity of quercetin by the colorimetric method.

To understand the relationship between the two methods, a plot was drawn with the measured values of quercetin from 11 silkworm cocoons in Table 3 by the HAE-HPLC method as an abscissa and the colorimetric method as an ordinate (Fig. 4). The plot shows the correlation between the total flavonoids detected by the HAE-HPLC and colorimetric methods. Correlation coefficients (*R*) were suggested to analyze the relationship between the total flavonoids detected by HAE-HPLC and the colorimetric method using Origin (8.5) software. The data showed almost no correlation (*R* = 0.62) existed between the two methods. In other words, the analysis and determination of the total flavonoids from silkworm cocoons or similar biomaterials using this colorimetric method is incorrect. It does not truly reflect the amount of total flavonoids existing in the silkworm cocoons.

### Antioxidant activity of HAEs in vitro

**Fig. 5:**
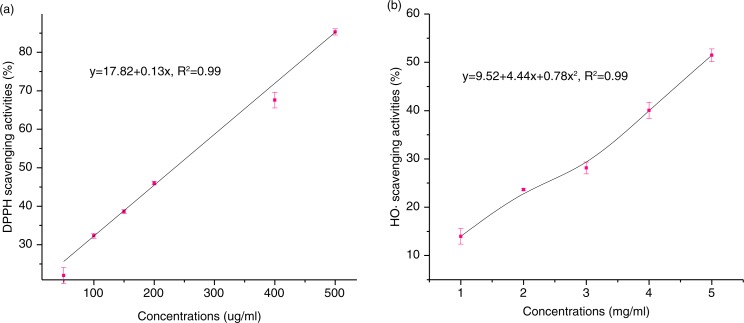
*In vitro* antioxidant effects of HAEs. (a) DPPH radical scavenging activities of HAEs, (b) HO· scavenging effects of HAEs.

As shown in Fig. 5, the common DPPH and HO· systems can be used to appraise the antioxidant potential of HAEs *in vitro*. The scavenging activity of HAEs on DPPH was 22, 32.34, 38.61, 46.01, 67.58, and 85.3% at various concentrations of 50, 100, 150, 200, 400, and 500 µg/mL (Fig. 5a). The IC_50_ value of DPPH scavenging activity is 243.63 µg/mL. Meanwhile, HAEs also showed the scavenging effects of 14.75, 21.54, 29.9, 39.81, and 51.3% against HO·at the concentrations 1–5 mg/mL (Fig. 5b). The IC_50_ value of HO· scavenging activity is 4.89 mg/mL. Therefore, the data indicated that HAEs had the ability to remove free radicals.

## Discussion

Silkworm cocoons are produced by a domesticated monophagous insect silk moth; its only food is mulberry leaves (23). The cocoon shell of the silkworm consists mainly of two proteins, fibroin and sericin. In addition to these proteins, the cocoon shell contains small amounts of pigments, waxes, and carbohydrates (12). The flavonols, especially quercetin, and their glycosides were present in mulberry leaves (24, 25). A glucosyl transferase in insects can modify the flavonoids and transfer a glucose residue to C-5 hydroxyl position of quercetin. This transformation can increase fitness and help increase the antioxidative state of the tissue (26). The flavonol aglycones also combine with other groups, such as amino acids (14). There are various flavonoid derivatives in a cocoon. Until now, these flavonol glycosides, which were known to exist in silkworm cocoons, were mostly quercetin glycosides and a few kaempferol glycosides. From our experimental results herein, there is a great difference in the type and content of flavonoids. A way to accurately determine the total flavonoids in the sericin layer of a silkworm cocoon is very important for new product development of these active substances in the medical biomaterials and function food industries. Recently, Guo et al. proposed some doubts about using the traditional colorimetric method that uses rutin as a standard to estimate the amount of total flavonoids in biosamples. Their results showed that those flavonoids, such as baicalin, baicalein, typhaneoside, kaempferol, quercetin, and hesperidin, have no maximum absorption at 500 nm. However, the non-flavonoids, such as caffeic acid, protocatechuic aldehyde, green chlorogenic acid, and protocatechuic, have maximum absorption (15). Our analytical results showed that the HAE-HPLC method described here has good reproducibility with an RSD (quercetin: 3.01% RSD and kaempferol: 2.38% RSD) and high recoveries of 99.56% (quercetin) and 99.17% (kaempferol). It could truly reflect the amount of total flavonoids that exist in silkworm cocoons. Moreover, almost no correlation (*R* = 0.62) exists between the values detected by the two methods for 11 silkworm cocoons. Therefore, the HAE-HPLC method constructed here can be used for truly estimating the total flavonoids through rapid quantitative analysis of the two aglycones, quercetin and kaempferol, which exist in silkworm cocoons or other functional foods. The hydrolysis-assisted extract exhibits that IC50 value of DPPH radical and HO· scavenging abilities are 243.63 µg/mL and 4.89 mg/mL, respectively. The HAEs can also be used for other antioxidant research *in vivo*.

## Conclusion

Dietary sericin possess anticancer, antihyperlipidemic, and antidiabetic activities. The established HAE-HPLC method is an efficient procedure for the hydrolysis of flavonol glycosides in the sericin layers of silkworm cocoons, extracting both aglycones, quercetin, and kaempferol, and rapidly measuring these aglycones quantitatively. The optimal hydrolysis of the flavonol glycosides and extraction of the aglycones occurs in a methanol–HCl–water (7:2:1, v/v/v) solution at 75°C over 4 h. The HAE-HPLC method has not only good reproducibility but also very high recoveries of quercetin and kaempferol. For 11 silkworm cocoons, there is almost no correlation (*R=*0.62) between the values determined by the HAE-HPLC method and the traditional colorimetric method using rutin as a standard.

## Supplementary Material

A new estimation of the total flavonoids in silkworm cocoon sericin layer through aglycone determination by hydrolysis-assisted extraction and HPLC-DAD analysis

## References

[1] Zhang YQ (2002). Applications of natural silk protein sericin in biomaterials. Biotechnol Adv.

[2] Rainer V (1993). Sericin silk protein: unique structure and properties. Cosmetics Toiletries.

[3] Sasaki M, Kato N, Watanabe H (2000). Inhibitory effect of silk protein, sericin on swelling and ulcerating of mouse large intestine. Oncol Rep.

[4] Yamade H, Fuwannomura M (1998). Use of sericin as antioxidants and tyroainase inhibitors. Eur Patent 0841065A2.

[5] Dash R, Mandal M, Kundu SC (2008). Silk sericin protein of tropical tasar silkworm inhibits UVB-induced apoptosis in human skin keratinocytes. Mol Cell Biochem.

[6] 
Sasaki M, Kato N, Watanabe H, Yamada H (2000). Suppresses colon carcinogenesis induced by 1, 2-dimethylhydrazine in mice. Oncol Rep.

[7] Toyosawat T, Terada S, Sasaki M (2006). Observation of individual cell behaviors to analyze mitogenic effects of sericin. Anim. Cell Technol Basic Appl A.

[8] Tsubouchi K, Igarashi Y, Takasu Y, Yamada H (2005). Sericin enhances attachment of cultured human skin fibroblasts. Biosci Biotechnol Biochem.

[9] Zhaorigetu S, Sasaki M, Watanabe H, Kato N (2001). Supplemental silk protein, sericin, suppresses colon tumorigenesis in 1, 2-dimethylhydrazine-treated mice by reducing oxidative stress and cell proliferation. Biosci Biotechnol Biochem.

[10] Okazaki Y, Kakehi S, Xu Y, Tsujimoto K, Sasaki M, Ogawa H (2010). Consumption of sericin reduces serum lipids, ameliorates glucose tolerance and elevates serum adiponectin in rats fed a high-fat diet. Biosci Biotechnol Biochem.

[11] Song CJ, Yang ZJ, Zhong MR, Chen ZH (2013). Sericin protects against diabetes-induced injuries in sciatic nerve and related nerve cells. Neural Regen Res.

[12] Tamura Y, Nakajimab KI, Nagayasua KI, Takabayashi C (2002). Flavonoid 5-glucosides from the cocoon shell of the silkworm, *Bombyx mori*. Phytochemistry.

[13] Kurioka A, Yamazaki M (2002). Purification and identification of flavonoids from the yellow green cocoon shell (Sasammayu) of the silkworm *Bombyx mori*. Biosci Biotechnol Biochem.

[14] Hirayama C, Ono H, Tamura Y, Nakamura M (2006). C-prolinylquercetins from the yellow cocoon shell of the silkworm. Phytochemistry.

[15] Guo YJ, Li F, Wang XQ, Zhang LZ (2002). Discussion about NaNO_2_ -Al(NO_3_)_3_-NaoH colorimetry for determination of total flavonoids. J Pharm Anal.

[16] Pensado L, Casais C (2000). Optimization of the extraction of polycyclic aromatic hydrocarbons from wood samples by the use of microwave energy. J Chromatogr A.

[17] Chen J, Wei JH, Li B, Cai SF, Zhang MZ (2012). Determination of contents of total flavonoids in cardiosrersum halicacabum by UV. Chin J Exp Tradit Med Form.

[18] Chen QC, Wang L, Liu Z (2008). Determination of quercetin in Polygonum capitatum by HPLC. PLA Pharm J.

[19] Zhao JG, Yan QQ, Zhang YQ (2014). Isolation and identification of colourless caffeoyl compounds in purple sweet potato by HPLC-DAD–ESI/MS and their antioxidant activities. Food Chem.

[20] Zhang M, Pan LJ, Jiang ST, Mo YW (2016). Protective effects of anthocyanins from purple sweet potato on acute carbon tetrachloride-induced oxidative hepatotoxicity fibrosis in mice. Food Agr Immunol.

[21] Celestino SB, Gary W (2003). Methods in polyphenol analysis.

[22] Lu LZ, Zhou YZ, Zhang YQ, Ma YL, Zhou LX (2010). Anthocyanin extracts from purple sweet potato by means of microwave baking and acidified electrolysed water and their antioxidation *in vitro*. Int J Food Sci Technol.

[23] Kaskoos RA, Ali M, Naquvi KJ (2012). Phytochemical investigation of the silk cocoons of *Bombyx mori* L. Int Res J Pharm.

[24] Kim GN, Jang HD (2011). Flavonol content in the water extract of the mulberry (*Morus alba* L.) leaf and their antioxidant capacities. J Food Sci.

[25] Sugiyama M, Katsube T, Koyama A, Itamura H (2013). Varietal differences in the flavonol content of mulberry (*Morus* spp.) leaves and genetic analysis of quercetin 3-(6-malonylglucoside) for component breeding. J Agric Food Chem.

[26] Hirayama C, Ono H, Tamura Y, Konno K, Nakamura M (2008). Regioselective formation of quercetin-5-O-glucoside from orally administered quercetin in silkworm. Phytochemistry.

