# Supplementary figures and images for: A new estimation of the total flavonoids in silkworm cocoon sericin layer through aglycone determination by hydrolysis-assisted extraction and HPLC-DAD analysis
Source: Food Nutr Res. 2016 Mar 14;60:10.3402/fnr.v60.30932. doi: 10.3402/fnr.v60.30932 (PMC4793258; doi:10.3402/fnr.v60.30932)

Supplementary Figure 1

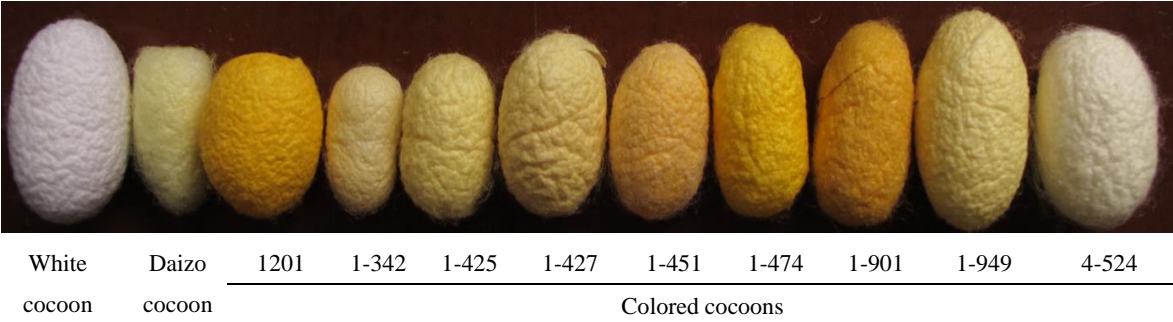

Supplement: A new estimation of the total flavonoids in silkworm cocoon sericin layer through aglycone determination by hydrolysis-assisted extraction and HPLC-DAD analysis [file FNR-60-30932-s001.pdf]
